# Gastric amyloidosis in a patient with dysphagia 

**Published:** 2021

**Authors:** Sami Ghazaleh, Anay Hindupur, Christian Nehme, Yasmin Khader, Marcel Ghanim, Taha Sheikh, Tarik Alhmoud

**Affiliations:** 1 *Department of Internal Medicine, University of Toledo Medical Center, Toledo, Ohio, USA*; 2 *Department of Gastroenterology, ProMedica Toledo Hospital, Toledo, Ohio, USA*

**Keywords:** Multiple myeloma, Plasma cell disorders, Malignancy, Amyloidosis, Dysphagia

## Abstract

Dysphagia is a symptom with a broad differential diagnosis. Usually, the etiology of dysphagia is benign, but it is essential to rule out serious causes. It is also critical to think outside the box when the etiology is not obvious. Herein, we describe a case of multiple myeloma that initially presented with dysphagia.

An 81-year-old male patient presented with dysphagia to solid food associated with anorexia and weight loss of 22 kg over the last 6 months. The patient looked chronically ill and cachectic. Upper endoscopy showed patchy erythema in the gastric antrum. Gastric biopsy was consistent with gastric amyloidosis. Although serum and urine protein electrophoresis did not show a monoclonal (M) band, immunofixation did show elevated free kappa light chains and elevated free Kappa/Lambda ratio. Bone marrow biopsy was consistent with multiple myeloma. Although gastrointestinal involvement is common in amyloidosis, it is unusual for amyloidosis to initially present in the gastrointestinal tract. Identification and treatment of the underlying condition, e.g., multiple myeloma, can lead to regression of gastrointestinal amyloidosis.

## Introduction

 Dysphagia is an alarming symptom in elderly patients that requires prompt medical investigation. While often seen as a common complaint, dysphagia may occur with various etiologies. Common causes for dysphagia in elderly patients include gastroesophageal reflux disease, benign peptic or postoperative stenosis, scleroderma, and Parkinson’s disease (1). Dysphagia in the elderly is also concerning for underlying malignancy causing mechanical obstruction or pseudoachalasia (2, 3). In this report, we describe a case of multiple myeloma that presented in an elderly male patient with dysphagia. 

## Case report

An 81-year-old man presented to the gastroenterology clinic complaining of progressive dysphagia to solid food for 6 months. He had poor appetite and weight loss of 22 kg. He denied chest pain, regurgitation, cough, abdominal pain, nausea, vomiting, diarrhea, hematochezia, or melena. His past medical history was significant for essential hypertension, coronary artery disease, chronic hepatitis B infection, hypothyroidism, and sarcoidosis. The patient’s past surgical history was significant for coronary artery bypass grafting (CABG) surgery, bilateral cataract surgery, and multiple tooth extractions. Family history was noncontributory. Home medications included aspirin, quinapril, atenolol, levothyroxine, and entecavir. He denied using tobacco, alcohol, or illicit drugs. 

On physical examination, the patient appeared chronically ill and cachectic. Vital signs demonstrated a temperature of 36.8 °C, blood pressure of 128/60 mmHg, heart rate of 65 beats per minute, and respiratory rate of 12 breaths per minute. Cardiovascular and lung exams were unremarkable. Abdominal exam showed a soft and non-tender abdomen with normal bowel sounds. Complete blood count (CBC) revealed a low hemoglobin of 11.6 g/dL and a mean corpuscular volume (MCV) of 63 fL consistent with microcytic anemia. Otherwise, the patient had a normal white blood cell (WBC) of 6.6 × 10^9^/L and platelets of 167 × 10^9^/L. Comprehensive metabolic panel (CMP) was within normal limits: Sodium 143 mmol/L, potassium 3.8 mmol/L, chloride 106 mmol/L, CO2 25 mmol/L, glucose 121 mg/dL, creatinine 0.81 mg/dL, BUN 12 mg/dL, calcium 9.4 mg/dL, total protein 6.5 g/dL, albumin 4.2 g/dL, total bilirubin 1.0 mg/dL, AST 12 U/L, ALT 12 U/L, and alkaline phosphatase 100 U/L.

**Figure 1:**
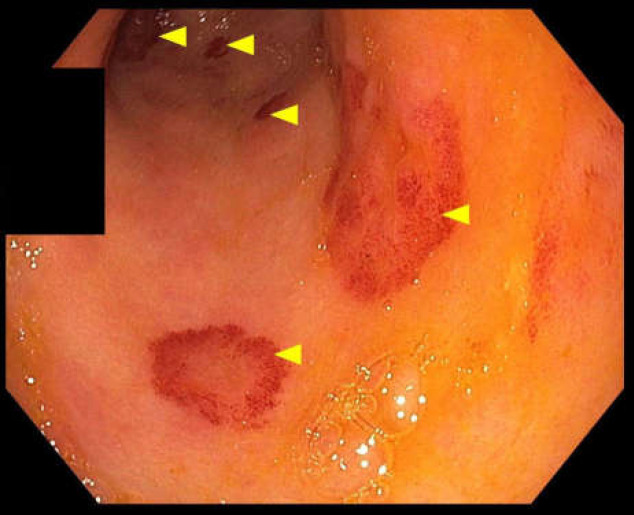
Esophagogastroduodenoscopy revealing patchy erythema in the gastric antrum (yellow arrows).

An esophagogastroduodenoscopy (EGD) was performed to investigate the cause of dysphagia. EGD revealed a normal appearing esophagus, patchy erythema in the gastric antrum, normal appearing gastric body, and normal appearing duodenum (Figure 1). A gastric biopsy was obtained, and histologic examination revealed glandular atrophy with acellular, eosinophilic deposits in the lamina propria and submucosa. Congo red stain revealed apple-green birefringence on polarized light consistent with gastric amyloidosis. 

Further workup followed to evaluate the etiology of amyloidosis. Serum protein electrophoresis (SPEP) did not show a monoclonal (M) band. Urine protein electrophoresis (UPEP) did reveal the presence of proteinuria, but also failed to show an M band. Immunofixation showed elevated free kappa light chains of 104.90 mg/dL and an elevated free Kappa/Lambda ratio of 156.57. At this point, the patient was referred to a hematology/oncology specialist for a bone marrow biopsy. Bone marrow biopsy showed that plasma cells constituted 28.5% of the bone marrow consistent with multiple myeloma. The patient was started on lenalidomide, bortezomib, and dexamethasone. One month later, he was seen for a follow-up visit at the oncologist’s office. He reported improvement in dysphagia and weight gain of 1 kg. Unfortunately, the patient’s malignancy progressed, and his condition continued to deteriorate over the next 5 months. He eventually developed pneumonia complicated by septic shock, respiratory failure, and acute kidney injury. He was admitted to the intensive care unit where he suffered a sudden cardiac arrest and expired.

## Discussion

Multiple myeloma is a plasma cell proliferative disorder commonly seen in the elderly population. It is characterized by the infiltration of plasma cells into the bone which causes skeletal disruption, bone pain, and hypercalcemia. Malignant plasma cells can secrete an M protein which leads to increased serum protein concentration and kidney dysfunction (4). SPEP is the initial test of choice for the detection of M protein which demonstrates an M band in about 82% of patients with multiple myeloma (4). However, it occasionally fails to detect an M band in patients who have only light immunoglobulin chains in their serum and urine. These patients are labeled as having light chain myeloma which accounts for 15-20% of multiple myeloma patients (5). This population can be detected by serum free light chain (FLC) assay, serum immunofixation, UPEP, and/or urine immunofixation. The combined use of these tests increases the sensitivity of M protein detection to 97% (4). The diagnostic criteria for multiple myeloma require a bone marrow biopsy consisting of at least 10% plasma cells and evidence of at least one of the major symptoms: lytic bone lesions, hypercalcemia, renal dysfunction, or anemia (6). However, the degree to which these symptoms appear is variable among patients. Signs of bone pain and anemia are seen in up to 58% and 73% of cases, respectively, whereas hypercalcemia and elevated creatinine are only seen in up to 28% and 48% of cases, respectively (4). This variation makes proper diagnosis of multiple myeloma challenging and further complicates it with the inclusion of other minor symptoms. Among these minor findings in multiple myeloma, the presence of light chain (AL) amyloidosis, only seen in 10-15% of patients, stands out for its ability to produce a wide array of atypical clinical signs (7).

In AL amyloidosis, amyloid proteins may deposit in various body organs causing great variation in clinical manifestations. This multiorgan system involvement may lead to excessive laboratory testing or misdiagnosis. For example, a reported case of AL amyloidosis induced cardiomyopathy has been described as a complication of multiple myeloma (8). This study highlights the difficulty of diagnosing multiple myeloma in the setting of AL amyloidosis as the major symptom of anemia seen in both oncologic and cardiovascular diseases (9).

While it has been reported that AL amyloidosis can cause dysphagia with involvement of the pharyngeal cavity and the tongue, dysphagia can also present due to amyloid deposition further down the gastrointestinal tract (10). AL amyloid deposition in the gastric mucosa can lead to nonuniformity in the lining of the stomach as has been shown in the small intestine (11). This discrepancy in stomach volume serves as an obstruction to the passage of gastric content and thus impedes swallowing food and liquid. Only a handful of cases have reported patients who presented with gastric outlet obstruction due to amyloidosis (12, 13). Our case had gastric involvement with erythematous amyloid deposits in the gastric antrum in addition to involvement of the oropharynx probably through amyloid myopathy (14).

It is common for AL amyloidosis to involve the gastrointestinal tract (15). However, it is unusual for patients with occult AL amyloidosis to present with gastrointestinal complaints. Patients can present with dysphagia, constipation, malabsorption, gastrointestinal bleeding, or protein-losing gastroenteropathy. Identifying the underlying etiology such as multiple myeloma is essential, because treatment can improve gastrointestinal amyloidosis.
